# A Simple Method for Transportation of Mouse Embryos Using Microtubes and a Warm Box

**DOI:** 10.1371/journal.pone.0138854

**Published:** 2015-09-22

**Authors:** Mikiko Tokoro, Noritaka Fukunaga, Kaori Yamanaka, Fumiaki Itoi, Yukari Terashita, Yuko Kamada, Sayaka Wakayama, Yoshimasa Asada, Teruhiko Wakayama

**Affiliations:** 1 Asada Institute for Reproductive Medicine, Asada Ladies Clinic Medical Corporation, Kasugai, Aichi, Japan; 2 Laboratory for Mouse Genetic Engineering, Quantitative Biology Center RIKEN, Suita, Osaka, Japan; 3 Kanazawa University School of Medicine, Kanazawa, Ishikawa, Japan; 4 Faculty of Life and Environmental Sciences, University of Yamanashi, Kofu, Yamanashi, Japan; 5 Advanced Biotechnology Center, University of Yamanashi, Kofu, Yamanashi, Japan; University of Connecticut, UNITED STATES

## Abstract

Generally, transportation of preimplantation embryos without freezing requires incubators that can maintain an optimal culture environment with a suitable gas phase, temperature, and humidity. Such incubators are expensive to transport. We reported previously that normal offspring were obtained when the gas phase and temperature could be maintained during transportation. However, that system used plastic dishes for embryo culture and is unsuitable for long-distance transport of live embryos. Here, we developed a simple low-cost embryo transportation system. Instead of plastic dishes, several types of microtubes—usually used for molecular analysis—were tested for embryo culture. When they were washed and attached to a gas-permeable film, the rate of embryo development from the 1-cell to blastocyst stage was more than 90%. The quality of these blastocysts and the rate of full-term development after embryo transfer to recipient female mice were similar to those of a dish-cultured control group. Next, we developed a small warm box powered by a battery instead of mains power, which could maintain an optimal temperature for embryo development during transport. When 1-cell embryos derived from BDF1, C57BL/6, C3H/He and ICR mouse strains were transported by a parcel-delivery service over 3 days using microtubes and the box, they developed to blastocysts with rates similar to controls. After the embryos had been transferred into recipient female mice, healthy offspring were obtained without any losses except for the C3H/He strain. Thus, transport of mouse embryos is possible using this very simple method, which might prove useful in the field of reproductive medicine.

## Introduction

In general, mammalian embryos are transported under cryopreserved conditions using liquid nitrogen or dry ice. Cryopreservation is suitable for the long-term preservation or transportation of embryos, but sophisticated laboratory apparatus is required to perform embryo freezing, and care is needed in shipping frozen embryos safely because of the hazards of using liquid nitrogen. By contrast, when live embryos are transported, freezing and thawing are not required. However, only short-term transportation is feasible with such embryos. When embryos are transported without freezing, a large and heavy incubator is required to maintain the humidity, temperature, and gas phase for embryo culture during transport. Therefore, new dedicated incubators suitable for mammalian embryo culture have been developed in recent years. For example, portable benchtop or desktop incubators have been used successfully to culture human embryos [1]. However, such incubators are bulky and require considerable installation space. Vajta et al. developed a culture system for bovine preimplantation embryos in which the culture dish was placed into a foil bag and submerged in a water bath instead of being placed in a CO_2_ incubator (“submarine incubation system”) [2]. However, the quality of the blastocysts was reduced and the number of blastomeres was decreased compared with standard incubators [3].

Recently, we reported the use of a simple incubator-free culture system with a deoxidizing agent [4]. In this system, most mouse embryos developed into blastocysts in a plastic bag without the need for maintaining humidity, and healthy offspring were obtained. Moreover, when the optimal temperature was maintained on a hot plate, the embryos could develop into blastocysts on the benchtop instead of in a CO_2_ incubator. Previously, we had considered that it would be important to maintain stability in all culture conditions because mammalian embryos are very sensitive to their culture environment. However, these studies suggested that mouse zygotes are more resilient to their environment than generally believed, and that if the temperature and gas phase can be maintained, embryos can develop into blastocysts anywhere using a simple system. Although, our system for embryo culture is very simple compared with the standard incubator-based system, embryos held in a culture dish are not stable for transportation because the dishes are not tightly sealed. If we could develop a system with a tight seal, it might be possible to transport embryos simply and at low cost by avoiding expensive apparatus and standard culture dishes.

An embryo culture method using a plastic dish covered with sterile paraffin oil was developed by Brinster [5]. This method can prevent the medium from evaporating without disturbing the gas exchange and allows observation of embryos because the oil is transparent. So far, several different culture methods have been reported [6–8], and the rate of development to the blastocyst stage in each method was similar to that found using Brinster’s method. However, the convenience of this culture system, including easy handling of embryos, low cost, and their high rate of development to the blastocyst stage, has led to wide adoption of the method. Observation is not required for transportation of embryos, but a tight seal is needed to prevent spillage of the medium. Roh et al. [8] reported that parthenogenetic mouse embryos could develop to blastocysts when cultured in polymerase chain reaction microtubes. Although they used a CO_2_ incubator and did not examine the potential for full-term development of the embryos, this suggested the possibility of using a tightly sealed microtube instead of culture dishes for transportation of embryos.

Here, we developed a simple, low-cost embryo transportation system using microtubes and a small warm box. We analyzed the rate of development of embryos cultured using this system, the quality and/or gene expression of the blastocyst, and full-term development because the strongest evidence of good-quality embryos is the production of live offspring. Finally, to test the system in practice, we transported mouse embryos from several strains between three cities (Kofu, Kobe, and Nagoya, Japan) using a parcel-delivery service without any special precautions, and examined the potential for the embryos to develop to full term.

## Materials and Methods

### Animals

Female and male B6D2F1 (C57BL/6J × DBA/2), C57BL/6J, C3H/He and ICR mice (8–12 weeks old) were obtained from the Shizuoka Laboratory Animal Center (Hamamatsu, Japan). On the day of the experiments—or after having finished all experiments—mice were euthanized by CO_2_ inhalation or by cervical dislocation and used for experiments. All experiments were conducted according to the Guide for the Care and Use of Laboratory Animals and were approved by the Institutional Committee of Laboratory Animal Experimentation of Yamanashi University.

### Production of zygotes by in vitro fertilization (IVF)

Spermatozoa were collected from the cauda epididymidis of male mice (>12 weeks) into 200 μL of Toyoda–Yokoyama–Hoshi (TYH) medium [9] (Mitsubishi Kagaku Iatron, Tokyo, Japan), or human tubal fluid (HTF) medium [10] covered with sterile mineral oil and capacitated by incubation for 1–2 h at 37°C under 5% CO2 in air. During sperm preincubation, cumulus–oocyte complexes (COCs) were collected from the oviducts of female mice (8–12 weeks old) that were induced to superovulate by consecutive injections of equine chorionic gonadotropin (5 IU) and human chorionic gonadotropin (5 IU) 48 h apart. Sixteen hours after the human chorionic gonadotropin injection, the mice were euthanized to collect COCs. After sperm preincubation, 5 μL aliquots of the suspension were added to droplets of HTF medium containing COCs. The final sperm concentration of this method was about 2 × 105 cells/mL. At 1.5 h after IVF, cumulus cells were dispersed by brief treatment with hyaluronidase (Type-IS, 150 units/mL; Sigma-Aldrich, St Louis, MO, USA). Fertilized zygotes, judged by extrusion of a second polar body, were collected from the droplets and washed in potassium simplex optimized medium (KSOM) [11] (Millipore, Billerica, MA, USA) or Chatot–Ziomek–Bavister (CZB) medium [12]. The zygotes were placed in fresh droplets of KSOM or CZB medium preincubated at 37°C under 5% CO2 in air and cultured for subsequent experiments. Control embryos were cultured in a culture dish continuously without being transferred into microtubes.

### Preparation of microtubes

**Fig 1:**
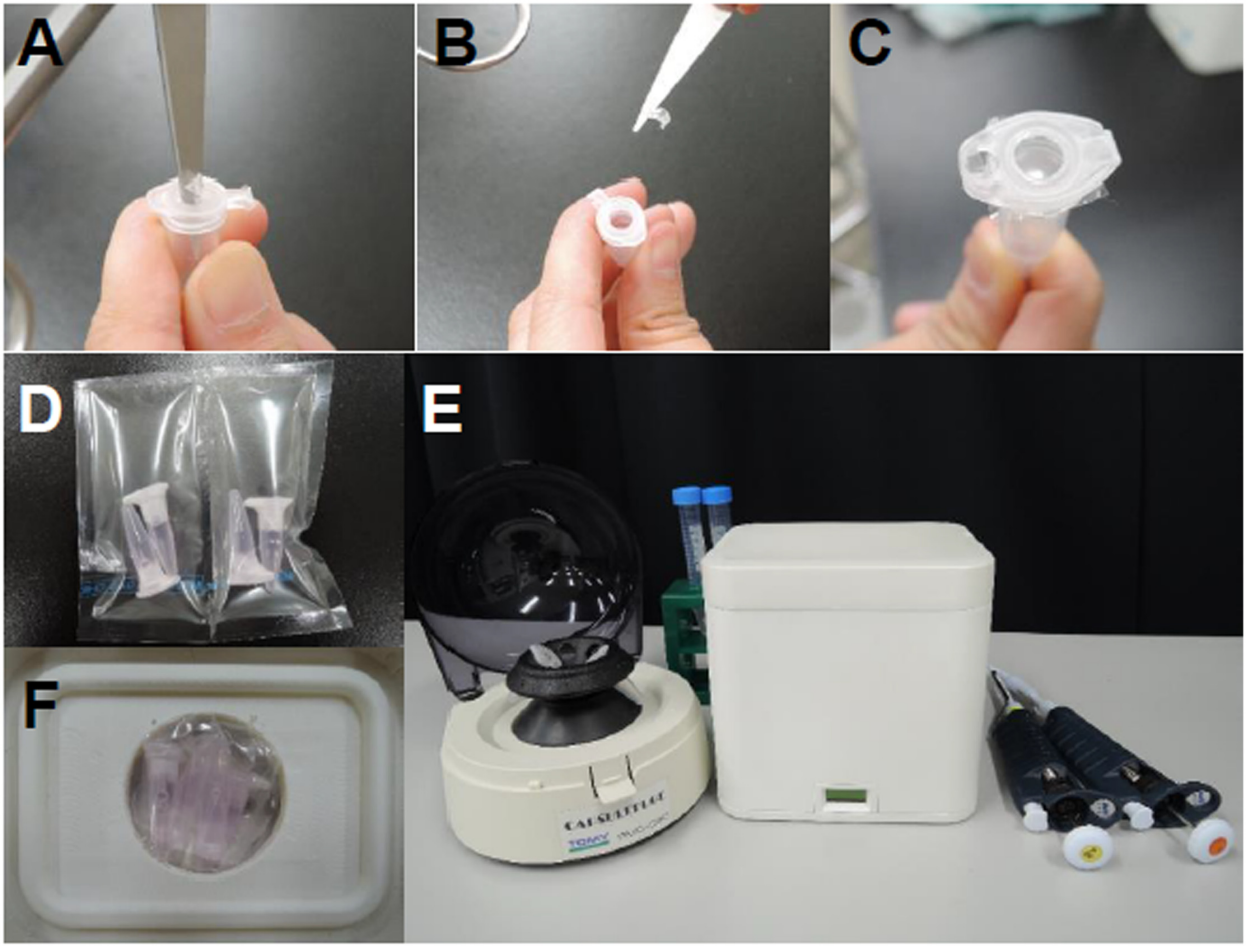
Microtubes and the simple warm box. (A) Puncturing a microtube lid using scissors. (B) A hole in the microtube lid facilitates gas exchange. (C) A microtube capped with a gas-permeable film. (D) Tubes were transferred into a plastic bag with the special gas mix. (E) Side view of the warm box showing a temperature indicator in the center. (F) A plastic bag placed in the box.

For this study, we prepared three different volume microtubes, 0.2 mL (tube A), 0.5 mL (tube B), and 1.5 mL (tube C). Each microtube had a hole in the lid to facilitate gas exchange. The microtube lid was punctured using scissors (Fig 1A and 1B), and the hole was covered using a gas-permeable membrane (Corefront Corporation, Tokyo, Japan; Fig 1C). The microtubes were washed by filling them with culture medium and placing them in a 5% CO_2_ incubator at 37°C overnight, and then the culture medium was discarded. The volumes of the microtubes were 1800 μL (tube A), 717 μL (tube B) and 333 μL (tube C). The empty microtubes were refilled completely with fresh culture medium, placed in the incubator for at least 6 h to equilibrate with the gas phase, and then used. A trial experiment was done using unwashed microtubes. As a result, all later experiments used Type B tubes (Sarstedt, Nümbrecht, Germany).

### Embryo culture in microtubes with or without a gas-permeable film seal

Pronuclear-stage zygotes were transferred into microtubes (30–60 in each tube) filled with culture medium, with or without a gas-permeable film. The microtubes were tightly sealed with Parafilm M (Pechiney Plastic Packaging, Chicago, IL, USA) and cultured either in a plastic bag (Mitsubishi Gas Chemical Co, Tokyo, Japan) filled with a gas mixture (5% CO_2_, 5% O_2_) or in a 5% CO_2_ incubator. Although the gas phases differed between experiments and control culture systems, a 5% CO_2_ incubator is commonly used in embryology laboratories and is cheaper than a 5% CO_2_, 5% O_2_ incubator. Moreover, we could introduce any mix of gas into the plastic bag from a syringe with minimal cost. Therefore, for practical purposes, we decided to use these conditions for the study. The plastic bag measured 9 cm × 9 cm and contained one to four microtubes (Fig 1D). The pH of culture media collected from tubes or control dishes was measured before and after embryo culture (4 days).

### Development of a warm box

To transport embryos, we developed a warm box (Fig 1E). We minimized the size and weight as much as possible, and powered it with a rechargeable battery. The box measured 15.4 × 14.8 × 12.4 cm and weighed 635.1 g (996.6 g with batteries). The storage space in the box was 4.0 cm across and 1.8 cm deep (Fig 1F). The temperature was set at 38 ± 1°C to maintain optimal culture conditions irrespective of the outside temperature. The box can maintain this temperature for more than 4 days.

### Transportation of embryos using a warm box

Zygotes derived from IVF at 6 h after insemination were transferred to microtubes and packed into a plastic bag filled with the gas mix. The bag was placed in the warm box, and sent by a parcel-delivery service from Kobe to Nagoya and then from Nagoya to Kobe (an approximately 400 km round trip), or from Kofu to Nagoya and then from Nagoya to Kofu (an approximately 500 km round trip). After the return to Kobe or Kofu, 4 days after IVF, the developing embryos were checked and transferred to recipient pseudopregnant female mice.

### Embryo transfer

Morula/blastocyst-stage embryos derived from embryos cultured in microtubes were transferred into the uterus of a pseudopregnant ICR female mouse at 2.5 days postcopulation (dpc). These mice had been mated with vasectomized ICR males. On the day of embryo transfer, recipients were anesthetized by intraperitoneal injections of 0.02 mL/g of tribromoethanol. Five to 10 embryos were transferred into each uterine horn [4,13]. Offspring were obtained at 18.5 dpc via Cesarean section, and the body and placental weights were recorded. In few experiments, Cesarean sections were performed at 16.5 dpc. Selected offspring were fostered to another ICR female mouse to allow them to grow to adulthood. When they matured sexually, randomly selected male and female offspring were paired and mated to test their fertility.

### Immunostaining

To count the total numbers of nuclei, and the numbers of trophectoderm (TE) cells and inner cell mass (ICM) blastomeres, immunofluorescence staining of blastocysts was performed as described [13]. The primary antibodies used were an anti-CDX2 mouse monoclonal antibody (1:200; MU392A0812X, Lot# MU392A0812; BioGenex, San Ramon, CA, USA) to detect the TE cells and an anti-Oct3/4 rabbit polyclonal antibody (1:100; sc-9081, Lot#L2211; Santa Cruz Biotechnology, Tokyo, Japan) to detect the ICM cells. The secondary antibodies used were Alexa Fluor 488-labeled goat anti-mouse IgG (1:500 dilution; Molecular Probes, Eugene, OR, USA) and Alexa Fluor 564-labeled goat anti-rabbit IgG (1:500 dilution; Molecular Probes). DNA was stained with 4',6-diamidino-2-phenylindole (DAPI; 2 μg/mL; Molecular Probes).

### Statistical analysis

Outcomes were evaluated using χ^2^ tests and *P* < 0.01 or < 0.05 were regarded as statistically significant.

## Results

### Effect of washing microtubes on embryo development

**Fig 2:**
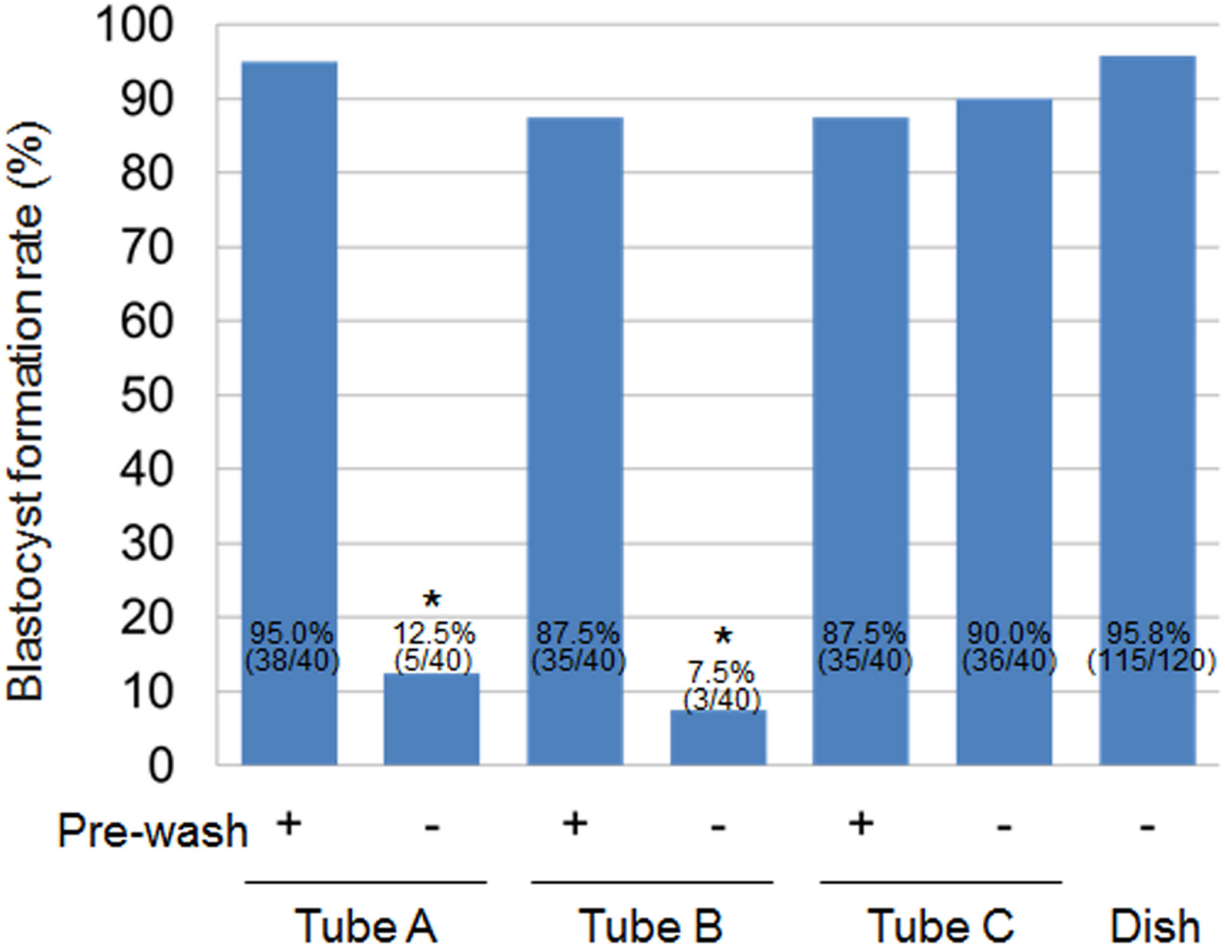
The rates of blastocyst development from zygotes cultured in three different types of microtubes with or without washing. Rates of development to the morula/blastocyst stages were examined at 72 h after insemination. Key: +, microtubes were washed with KSOM before embryo culture; –, microtubes were used without washing; dish, embryos were cultured in a culture dish by conventional methods as controls. *P < 0.05 by χ^2^ test.

When BDF1 mouse embryos were cultured in microtubes without a prewash step, the rates of morula/blastocyst formation in tubes A or B were significantly lower than in controls (12.5% and 7.5%, respectively; *P* < 0.01; Fig 2). However, when the microtubes were washed before embryo culture, most embryos reached the morula/blastocyst stage (87.5%–95.0%; Fig 2) with similar rates to the control embryos cultured in a culture dish (95.8%; Fig 2). By contrast, mouse embryos cultured in tube C, either prewashed or unwashed, showed a very high rate of development to the morula/blastocyst stage. These results suggest that prewashing of the A or B microtubes was necessary to ensure optimal embryo development to the morula/blastocyst stage, and all the following experiments used prewashed microtubes. This experiment was repeated twice for each group.

### Effect of a gas-permeable film on the development of embryos cultured in microtubes

When BDF1 mouse embryos are cultured in microtubes, gas permeability during culture is lower than when using a culture dish. Therefore, we used a gas-permeable film, and examined its effect on the development of mouse embryos cultured in microtubes. As shown in Table 1, after culturing embryos for 72 h, most of them reached the morula/blastocyst stage in all experimental groups (83%–98%) with similar rates to controls cultured in a culture dish (98%). However, our success rates in producing offspring using gas-permeable films were higher than those achieved without using it (24%–47% vs. 18%–30%, respectively). In particular, when embryos were cultured in type B tubes with a gas-permeable film, the success rate was significantly higher than in the other experimental groups (*P* < 0.05; Table 1).

**Table 1:** Full-term development of embryos cultured in microtubes with or without a gas-permeable film.

Type of microtubes	Gas perm. film	No. embryos examined	No. 1–8 cell (%)	No. M/ B (%)	No. Frag. (%)	No. embryos transfered (recipients)	No. offspring (%)	No. implant. (%)
Tube A	+	40	3 (8)	34 (85)	3 (8)	34 (2)	8 (24)^a^	16 (47)
	–	40	3 (8)	33 (83)	4 (10)	33 (2)	6 (18)^a^	10 (30)
Tube B	+	40	1 (3)	38 (95)	1 (3)	38 (2)	18 (47)^b^	25 (66)
	–	40	1 (3)	37 (93)	2 (5)	37 (2)	11 (30)^a^	14 (38)
Tube C	+	40	1 (3)	38 (95)	1 (3)	38 (2)	13 (34)^a^	19 (50)
	–	40	0 (0)	39 (98)	1 (3)	39 (2)	8 (21)^a^	12 (31)
Dish	–	40	1 (3)	39 (98)	0 (0)	39 (2)	17 (44)^a^	24 (62)

Values with different superscript letters are significantly different (*P* < 0.05 by χ^2^ tests).

Key: Gas perm. film, gas-permeable film; M/B, Morula/Blastocyst; Frag, fragmentation; implant., implantation

**Fig 3:**
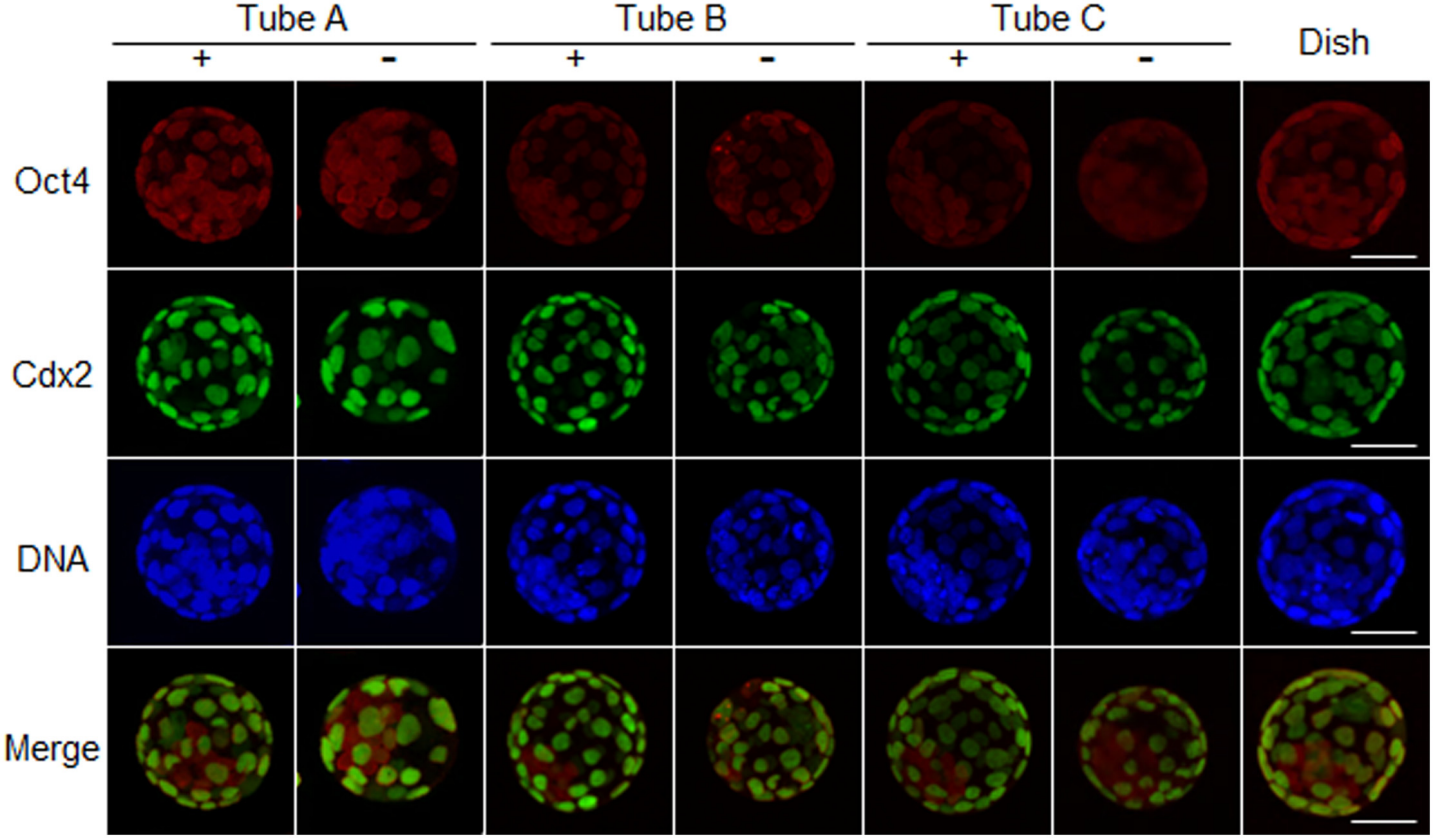
The quality of blastocysts after culture in microtubes with or without a gas-permeable film; immunostaining of blastocysts at 96 hours after IVF. The embryos were cultured in three different types of microtubes and then examined for Pou5f1 expression (red), a marker for the inner cell mass (ICM); for CDX2 expression (green), a marker for the trophectoderm (TE); and with DAPI (blue) to stain nuclei (DNA). Merged images are shown for Pou5f1 and CDX2. Key: +, embryos cultured in microtubes with a gas-permeable film;–, embryos cultured in sealed microtubes; dish, embryos grown in a culture dish using a conventional method as controls. Scale bar = 50 μm.

The quality of blastocysts cultured in microtubes with or without a gas-permeable film was evaluated based on the cell number and allocation of ICM cells, using immunostaining as shown in Fig 3. When embryos were cultured in tube A, B, or C without a gas-permeable film, the mean ICM and TE cell numbers (ICM 10.8, 14.0, and 17.9; TE 32.2, 34.6, and 43.3, respectively) were lower compared with embryos cultured in microtubes with a gas-permeable film (ICM 15.8, 20.6, and 20.4; TE 32.2, 34.6, and 43.3, respectively). However, when embryos were cultured in tube A, B, or C with a gas-permeable film, the mean total numbers of ICM and TE cells (70.3, 79.7, and 82.6, respectively) were similar to those in the controls (86.9). These results suggest that the use of a gas-permeable film improved the quality of blastocysts and the success rate of producing offspring derived from embryos cultured in microtubes. Moreover, type B tubes were better for culturing embryos than the type A and type C tubes. This experiment was repeated twice in each group (Table 1). Therefore, we used type B tubes for all the following experiments.

### Embryo culture in the warm box

We developed a simple warm box that could maintain an appropriate temperature to allow the transport of BDF1 mouse embryos without impairing their developmental potential (Table 2). When microtubes with or without a gas-permeable film were placed in a plastic bag filled with the special gas mixture and cultured in the warm box, the rate of morula/blastocyst formation in both microtubes was similar to that in control groups (96% vs. 99%; Table 2). The pH of the culture medium was also similar between experiment and control cultures (pH 8.1 vs. pH 8.1, respectively), and before and after embryo culture (pH 8.1 vs. pH 8.3, respectively). However, the rate of formation of live offspring derived from embryos cultured in microtubes without a gas-permeable film was lower than when using a gas-permeable film or in controls (25% vs. 36% or 37%, respectively. This experiment was repeated twice for each group; Table 2). All these mice grew to adulthood and were fertile.

**Table 2:** Full-term development of embryos cultured in the warm box.

Culture method	Gas perm. film	No. embryos examined	No. 1–8 cell (%)	No. M/ B (%)	No. Frag. (%)	No. embryos transfered (recipients)	No. offspring (%)	No. implant. (%)
Warm box	+	80	0 (0)	77 (96)	3 (4)	77 (4)	28 (36)	57 (74)
	–	80	0 (0)	77 (96)	3 (4)	77 (4)	19 (25)	39 (51)
Incubator	+	80	0 (0)	79 (99)	1 (1)	79 (4)	29 (37)	56 (71)

Key: Gas perm. film, gas-permeable film; M/B, Morula/Blastocyst; Frag, fragmentation; implant., implantation

### Transportation of mouse embryos using the warm box

**Fig 4:**
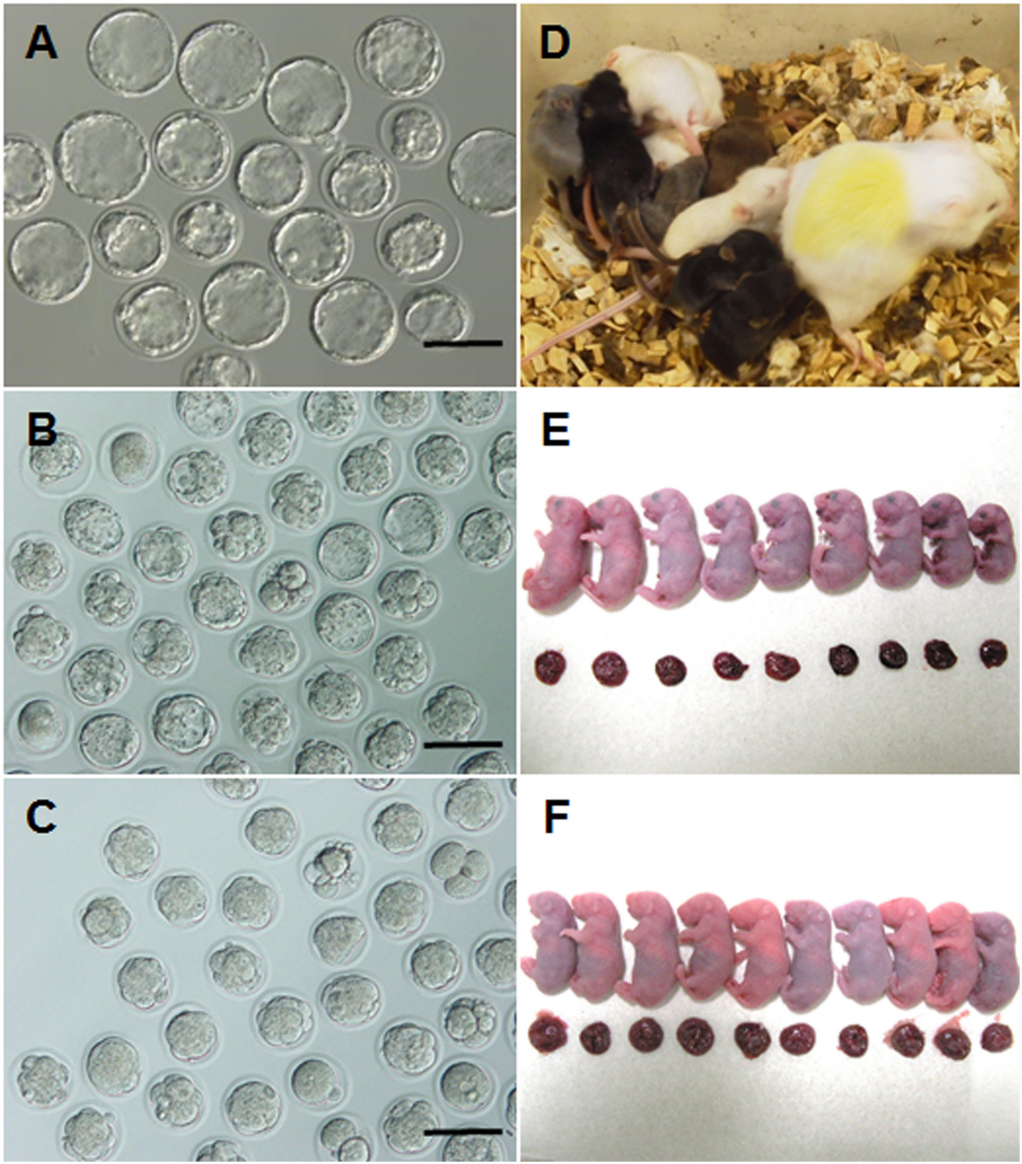
Development of full-term offspring derived from embryos transported by parcel-delivery service. (A–C) morulae/blastocysts from BDF1, C57BL/6 and ICR mouse strains, respectively, cultured in a warm box using microtubes with a gas-permeable film and placed in a plastic bag containing the special gas mixture, and then transported by parcel-delivery service. BDF1 mouse embryos were cultured for 96 h in the warm box, but the other embryos were cultured for 72 h. (D–F) Full-term offspring from BDF1, C57BL/6 and ICR mouse strains, respectively, were obtained through Cesarean section and fostered by another ICR female mouse. BDF1 offspring (colored pups in D) are shown at 1 week after birth. The other pups are shown just after Cesarean section.

To demonstrate the practical use of the warm box, a box containing pronuclear-stage BDF1 mouse embryos was transported via a parcel-delivery service from Kobe to Nagoya, and then Nagoya to Kobe (approximately 400 km for the round trip) without any special precautions. Although this round trip took only 2–3 days, the warm box was kept in the laboratory until the afternoon of day 4 following IVF (a total of 4 days culture in the box including transportation). After collecting the microtubes from the box, we found that most embryos had developed to the blastocyst stage in microtubes with or without a gas-permeable film (98% and 95%, respectively; Table 3 and Fig 4A). Although offspring were obtained from both groups after transfer into recipient female mice (Fig 4D), the success rate for the group cultured in tubes with the gas-permeable film was higher than that for the group cultured in tubes without a gas-permeable film (54% vs. 39%, *P* = 0.2063; Table 3). This experiment was repeated twice for each group.

**Table 3:** Full-term development of BDF1 mouse embryos transported by parcel-delivery service.

Gas perm. Film	No. embryos examined	No. 1–8 cell (%)	No. M/ B (%)	No. Frag. (%)	No. embryos transfered (recipients)	No. offspring (%)	No. implant. (%)	Mean body weight (g)	Mean placental weight (g)
**+**	40	0 (0)	39 (98)	1 (3)	39 (2)	21 (54)	38 (97)	1.25 ± 0.19	0.12 ± 0.03
**–**	40	0 (0)	38 (95)	2 (5)	38 (2)	15 (39)	26 (68)	1.32 ± 0.22	0.13 ± 0.03

Key: Gas perm. film, gas-permeable film; M/B, Morula/Blastocyst; Frag, fragmentation; implant., implantation

Finally, we examined other mouse strains, because the most prevalent strains used in research are either C57BL/6 or 129, and it is generally believed that the B6D2F1 strain has higher developmental competence than inbred strains. Pronuclear-stage C57BL/6, C3H/He, and ICR embryos derived from IVF were placed in the box and transported via a parcel-delivery service from Kofu to Nagoya and then Nagoya to Kofu (approximately 500 km round trip) without any special precautions. After transportation, embryos were collected from the microtubes. Although the rates of development to the morula/blastocyst stage were lower than with BDF1 embryos, good development rates to morula/blastocyst stages were obtained (60–64%), similar to the control experiment (zygote were cultured in 5% CO_2_ incubator at the same time; 51–80%). This experiment was repeated twice for each group (Table 4 and Fig 4). Offspring were obtained from C57BL/6 and ICR strain embryos after transfer into recipient female mice (Fig 4).

**Table 4:** Full-term development of inbred or outbred mouse strain embryos transported by parcel-delivery service.

Culture method	Gas perm. film	Mouse strains	No. of embryos examined	No. 1–8 cell (%)	No. M/ B (%)	No. Frag. (%)	No. embryos transferred (recipients)	No. offspring (%)	No. implant. (%)
Warm box	+	C57BL/6	112	25 (22)	67 (60)	20 (18)	67 (3)	12(18)	33(49)
	+	C3H/He	83	25 (30)	51 (61)	7 (8)	51 (4)	0(0)	0(0)
	+	ICR	105	20 (19)	67 (64)	18 (17)	44 (2)	16(36)	23(52)
Incubator (Control)	+	C57BL/6	117	40 (34)	60 (51)	17 (15)	45 (2)	14(31)	28(62)
	+	C3H/He	35	7 (20)	28 (80)	0 (0)	28 (1)	4(14)	14(50)
	+	ICR	86	19 (22)	48 (56)	19 (22)	48 (3)	18(38)	24(50)

Key: Gas perm. film, gas-permeable film; M/B, Morula/Blastocyst; Frag, fragmentation; implant., implantation

## Discussion

In this study, we successfully obtained healthy offspring from hybrid and inbred mouse strain embryos transported by a parcel-delivery service using culture in microtubes and our newly developed warm box. During this study, we found several important factors for successful culture of embryos.

We examined the types of microtubes and the effect of washing prior to embryo culture. When microtubes were used without washing, two of the three types of microtubes showed very poor embryo development to the blastocyst stage. However, when microtubes were used after washing, most embryos reached the morula/blastocyst stage at rates similar to those of control embryos cultured in dishes. These results suggest that these two types of microtubes (type A and type B tubes in this study) contain toxins affecting embryo development. It is well known that some polymers show detrimental effects on gametes and embryos [14]. Here, we used polypropylene microtubes, which are in general laboratory use and are certified as being free of RNase, DNase, and endotoxins. Whether it was the direct toxicity of the polypropylene or some kind of chemical added during the manufacturing process that affected the embryos is not clear. However, it is likely that the putative toxin is on the surface of microtubes rather than eluted from the polymer, because the blastocyst formation rate was significantly improved by just one wash. Type C tubes did not show any detrimental effects on embryo culture even without washing. Therefore, the toxicity of microtubes for embryo culture is not a problem if the tubes are carefully selected or washed before use.

Initially, we thought that efficient gas exchange would be essential for embryo culture. Therefore, gas-permeable films were placed on the punctured lids of microtubes, and embryos cultured in those microtubes showed a high rate of development similar to that seen with controls cultured in dishes that allow free gas exchange. Surprisingly, embryos cultured in sealed microtubes also developed to blastocysts. Although the rate of blastocyst formation from embryos cultured in this way was lower than when they were cultured in microtubes with a gas-permeable film, the findings suggest that gas exchange is not essential in this system. In this study, all microtube lids were closed tightly and sealed with Parafilm M. Even so, gas exchange might occur through the very small gap between the tube and the lid, and through the seal. Another possibility is that the embryo culture medium was prepared at least 6 h before the experiment to equilibrate it with the gas phase used. This medium might support embryo development without gas exchange for up to 4 days. Very recently, several simple embryo culture systems have been reported [4,15–17]. Although they share certain disadvantages, such as transportation difficulties, all reports suggest that mammalian embryos are more resilient to their environment than generally believed.

To transport embryos without freezing, they must arrive within a few days. When embryos are cultured at low temperatures, the preservation period can be extended by at least 2 days [18,19]. However, such cooling can embryos adversely [19–21]. By contrast, using the optimal temperature for embryo culture cannot extend the preservation period. To obtain high-quality embryos and many offspring after transportation, it is necessary to maintain an optimal temperature instead of cooling the culture for preservation.

Parcel-delivery services in Japan are quick, and usually 1, 2, or 4 days are sufficient for delivery of any parcel to any domestic location or even internationally. Embryos should be maintained in an optimal environment when using such a service for transport. In our previous study, we demonstrated that an optimal gas phase could be maintained using a plastic bag [4]. Here, we have developed a simple warm box that could maintain an optimal temperature for at least 4 days. When zygotes in microtubes were cultured in this box without using a plastic bag, embryo development was arrested completely (0/103 grew in a preliminary study, repeated twice). It is known that some embryos can develop to blastocysts without gas-phase control [4]. Therefore, the warm box probably volatilizes one or more substances detrimental to embryo development, and these substances might pass into the microtubes through the gap between the lid and the tube. However, in the current study, we used a plastic bag to maintain the gas phase. When embryos were cultured in this way, the plastic bag might have prevented any toxic gas from the box reaching the cultures, as well as maintaining an optimal environment for embryo development. Embryos cultured in this manner could develop into blastocysts and term offspring at rates similar to those of control embryos cultured in dishes. Moreover, if plastic bags are used for embryo culture, we can choose any gas mixture for injecting using a syringe, such as 5% CO_2_, 5% O_2_, and 90% N_2_. This also suggests that there is no need to purchase an expensive gas-controlling incubator. Thus, a system using microtubes, a plastic bag, and a warm box can support embryo development without any significant losses, nor the need for expensive equipment. We sent embryos in a parcel to a different city without any special precautions and normal offspring were obtained with a high success rate except for the C3H/He mouse strain, which demonstrates how practical this system is for embryo transportation. Our trial model of the warm box is already very small, but we might be able to develop an even smaller box in the future. Although we failed to obtain C3H/He offspring from this transportation method, it was because the recipient mice in this experiment were not optimally conditioned because the in vitro development of C3H/He embryos were not affect.

To our knowledge, the warm box we developed is the simplest and smallest transport system yet available for live mouse embryos. This system does not require the embryo to be frozen and thawed. The transportation cost is low because of the small size of the box, which can be transported at ambient temperature. Because this system will pass through quarantine controls, embryo transport is more convenient than transporting live mice. If this transportation method could be used for the embryos of animals other than mice—such as dairy cattle—transporting embryos could enhance selective breeding. In addition, we believe that this system will be also applicable for overnight transportation of IVM and IVF oocytes and for SCNT embryos. Therefore, this system will be very useful as a simple and cost-effective method for embryo transport without the need for special precautions.
